# Selective Autophagy and Xenophagy in Infection and Disease

**DOI:** 10.3389/fcell.2018.00147

**Published:** 2018-11-13

**Authors:** Vartika Sharma, Surbhi Verma, Elena Seranova, Sovan Sarkar, Dhiraj Kumar

**Affiliations:** ^1^Cellular Immunology Group, International Centre for Genetic Engineering and Biotechnology, New Delhi, India; ^2^Institute of Cancer and Genomic Sciences, Institute of Biomedical Research, College of Medical and Dental Sciences, University of Birmingham, Birmingham, United Kingdom

**Keywords:** xenophagy, ubiquitination, p62, NDP52, OPTN, TAX1BP1, inflammation, DUBs

## Abstract

Autophagy, a cellular homeostatic process, which ensures cellular survival under various stress conditions, has catapulted to the forefront of innate defense mechanisms during intracellular infections. The ability of autophagy to tag and target intracellular pathogens toward lysosomal degradation is central to this key defense function. However, studies involving the role and regulation of autophagy during intracellular infections largely tend to ignore the housekeeping function of autophagy. A growing number of evidences now suggest that the housekeeping function of autophagy, rather than the direct pathogen degradation function, may play a decisive role to determine the outcome of infection and immunological balance. We discuss herein the studies that establish the homeostatic and anti-inflammatory function of autophagy, as well as role of bacterial effectors in modulating and coopting these functions. Given that the core autophagy machinery remains largely the same across diverse cargos, how selectivity plays out during intracellular infection remains intriguing. We explore here, the contrasting role of autophagy adaptors being both selective as well as pleotropic in functions and discuss whether E3 ligases could bring in the specificity to cargo selectivity.

## Introduction

Autophagy, a basal cargo degradation process, is responsible for elimination of superfluous and unwanted cytoplasmic materials including misfolded proteins and aggregates, damaged organelles and other macromolecules including lipids and carbohydrates in the cells. While basal autophagy is important for maintaining homeostasis by providing energy substrates to the cell, this process also gets induced by various environmental cues, including stress (osmotic, nutritional, serum starvation) and pathogen stimulation. The complexity of this seemingly simple process of cargo degradation began to be unraveled following the discovery of *ATG* family of genes in yeast cytoplasm to vacuole targeting (cvt) pathway (Nakatogawa et al., 2009). Since then, mammalian orthologs of yeast *Atg* genes have been found which perform similar functions but in a more sophisticated manner. The step-wise process of cargo tagging, autophagosome formation and targeting them to the lysosomes for degradation has been fairly well studied (Levine et al., 2011; Deretic, 2012; Feng et al., 2014; Mizushima, 2018; Yu et al., 2018).

In selective autophagy, specific cargos are first tagged by ubiquitination, following which they get recognized by the autophagy adaptor molecules for subsequent targeting to autophagosomes for degradation. The tagging and targeting of cargos imparts selectivity to the degradation process that is referred to as selective autophagy, which is different from bulk degradation of the packaged cargo that occurs in a non-selective manner. The selective cargos could be misfolded proteins, damaged organelles like mitochondria and peroxisomes or intracellular pathogens like *Mycobacterium* spp., *Salmonella* spp., *Listeria* spp. amongst others (Gatica et al., 2018). Based on the cargo being delivered for degradation, selective autophagy has been classified into mitophagy (degradation of damaged mitochondria), pexophagy (peroxisomes), lipophagy (lipid droplets), glycophagy (glycogen), ribophagy (ribosomes), ER-phagy (ER) and xenophagy (intracellular pathogens) (Gatica et al., 2018). Considering the diversity of potential targets autophagy could degrade, it is plausible that they are implicated in regulating diverse physiological processes including cellular homeostasis, inflammation as well as fate of intracellular infection. Nutrient recycling is one of the earliest discovered functions of autophagy, which helps maintain cellular homeostasis by extracting energy from catabolic substrates during energy requirement in diverse stress conditions including bacterial infections. This key homeostatic function of autophagy can be exploited by bacterial pathogens to source nutrients for their own survival, adding another dimension on how autophagy could impact bacterial survival during infections (Chaston and Goodrich-Blair, 2010).

Interestingly targeting of both intracellular cargos as well as intracellular pathogens rely on the core autophagic tagging, recognition and degradation machinery. Ubiquitination of cargos is among the first steps in targeting them toward autophagic degradation, which may be akin to an intracellular “eat-me” signal (Boyle and Randow, 2013; Shaid et al., 2013). Ubiquitinated cargos are subsequently recognized by autophagy adaptors (also called autophagy receptor proteins), which then tag them to phagophores, the nascent autophagic membranes, subsequently maturing into autophagosomes (Shaid et al., 2013; Stolz et al., 2014). It is therefore widely appreciated that shared components of autophagic machinery get involved irrespective of whether it is for the homeostatic purposes or for the cellular defense mechanisms. While each of the different possible autophagic cargos and the corresponding selective machinery involved have been extensively studied (Gatica et al., 2018), majority of these studies mostly rely on a particular kind of cargo in isolation. Under physiological conditions, especially during intracellular infections, however, different arms of autophagic machinery must work in conjunction considering the intertwining of homeostatic and defense functions of autophagy (Chandra and Kumar, 2016). How cargo tagging, recognition and degradation works in specific manner when multiple potential targets for autophagic degradation are present inside the cell, remains obscure. Specifically, during intracellular infections, where xenophagy occurs alongside selective autophagy for intracellular cargos, how ubiquitin ligases and autophagy adaptors discriminate among such cargos within same cell, remains unexplored. In this review, we try to bring together the selectivity and redundancy in the roles of different regulators in terms of cargo tagging, recognition and autophagy-mediated degradation during cellular homeostatic and defense functions.

## Autophagy as a Homeostatic and Anti-Inflammatory Cellular Process

Inflammation is a stress-mediated cellular response, elicited by infections or tissue damages and triggered by either cell-intrinsic or extrinsic factors (Chovatiya and Medzhitov, 2014). The surveillance machinery for initiating inflammatory responses involves pattern recognition receptors (PRRs), which recognizes pathogen-associated molecular patterns (PAMPs) or damage-associated molecular patterns (DAMPs) for pathogenic and cell-intrinsic factors respectively. Generally, signaling through PRRs eventually lead to formation of a large multi-molecular complex called inflammasome (Saitoh et al., 2009). The indication that autophagy could be involved in regulation of inflammation emerged first through a GWAS study on patients with Crohn’s disease (CD), an inflammatory disorder of the gut, where SNP in an autophagy-related gene *ATG16L1* show strong association with the disease susceptibility (Hampe et al., 2007). The physiological consequence of ATG16L1 function in regulating inflammation is evident in *ATG16L1* knockout animals, which show dramatically enhanced production of pro-inflammatory cytokines like IL-1β and IL-18 (Saitoh et al., 2008). Likewise, several studies report that increased inflammation during aging actually reflects loss of the autophagic capabilities resulting in accumulation of damaged, depolarized mitochondria (Green et al., 2011). These studies together highlight the homeostatic functions of autophagy. Other than aging, mitochondrial depolarization also acts as the trigger for activation of NLRP3 inflammasome. Accumulation of damaged mitochondria, and the resulting increase in cellular redox-stress upon inhibition of autophagy and mitophagy activates NLRP3 signaling leading to inflammasome activation (Zhou et al., 2011). Intriguingly, mitochondria are not the only cell organelle, which can directly impact inflammation. There are close associations reported between endoplasmic reticulum, peroxisomes, protein aggregates and inflammation (Schrader and Fahimi, 2006; Zhang and Kaufman, 2008; Gebbink et al., 2009; Garg et al., 2012). Interestingly, autophagy can selectively target each one of these organelles/cargos for degradation through reticulophagy or ER-phagy, pexophagy and aggrephagy, respectively. Thus autophagy, by virtue of its degradative capabilities, serves as a key anti-inflammatory pathway by selectively degrading components, which could potentially trigger inflammation.

**FIGURE 1:**
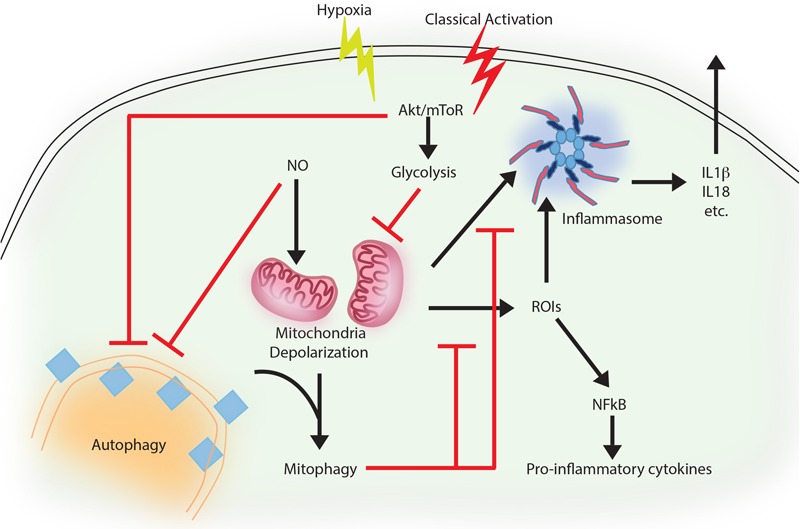
Autophagy as an anti-inflammatory process. Activation of macrophages upon IFNγ and LPS treatment results in classically activated macrophages, which are more phagocytic, microbicidal and inflammatory in nature. During classical activation, autophagy is inhibited in a NO-dependent manner. This coincides with mitochondrial depolarization and accumulation of such depolarized mitochondria due to lack of mitophagy (since the core machinery for autophagy and mitophagy are same). Depolarized mitochondria are source of heightened reactive oxygen intermediates (ROIs) in the activated macrophages and also activates NLRP3 mediated inflammasome pathway and secretion of cytokines like IL1β and IL18. Inhibition of autophagy occurs in mToR/Akt dependent manner and inhibition of Akt signaling can alleviate the autophagy levels and control mitochondrial depolarization by directing damaged mitochondria toward mitophagy. Together, inhibition of autophagy in these cells helps achieve the pro-inflammatory phenotype, establishing the anti-inflammatory function of autophagy. Similar events also occur when macrophages are exposed to hypoxia, with the exception of NO production, which does not occur under hypoxia.

Although, inflammation is the prime innate immune host response against any pathogen attack (Mogensen, 2009), prolonged inflammation may cause severe tissue damage (Saitoh et al., 2009; Qian et al., 2017; Takahama et al., 2018). Therefore, its regulation is important to check the prolonged effects of inflammation, including severe tissue damage and cell death. There is strong co-relation between autophagy and inflammation where on one hand autophagy supports the survival of inflammatory cells including macrophages, lymphocytes and neutrophils (Qian et al., 2017) and at the same time, it also controls the secretion of pro-inflammatory cytokines from innate immune effector cells (Qian et al., 2017). A positive role of autophagy has been described in suppressing mitochondrial reactive oxygen species (mtROS) and production of IL-1β, IL-18 in a p62, also called sequestosome 1 (SQSTM-1) dependent manner in response to rapamycin-induced autophagy in macrophages (Ko et al., 2017). Similar mechanism is also in display during pro-inflammatory stimulation of macrophages, where classically activated macrophages (M1 or the inflammatory sub-type) shows decline in autophagy, which allows these cells to acquire the inflammatory potential (Matta and Kumar, 2015). This process, schematically shown in Figure 1, to a large extent, is dependent on AKT mediated shift toward aerobic glycolysis (Matta and Kumar, 2015). This is also true during Toll Like Receptor (TLR) stimulation of Dendritic Cells (DCs) or macrophages, where inhibition of autophagy, due to loss of ATG16L1 or ATG7, causes massive pro-inflammatory cytokine signaling including IL-1β and IL-18 (Saitoh et al., 2008). Similarly, in the diabetic model of macrophages, autophagy inhibition exhibits generation of reactive oxygen species (ROS) and pro-inflammatory cytokines including IL-1β (Dai et al., 2017). An interesting mechanism of the anti-inflammatory effector function of autophagy revealed recently show that loss of autophagy protein ATG16Ll in macrophages results in the accumulation of adapter protein TRIF, which otherwise gets targeted for degradation through selective autophagy adaptors p62 and Tax-1 binding protein 1 (TAX1BP1). The study further shows knocking down of TAX1BP1 also help enhance pro-inflammatory cytokine signaling, further establishing the role of selective autophagy in limiting inflammation (Samie et al., 2018).

Priming of macrophages with IFNγ helps controlling *Mycobacterium tuberculosis* via increased maturation of phagosomes in IRGM mediated manner (Singh et al., 2006). IRGM is a strong mediator of inflammation and regulates secretion of IL-1β, IL-4, IL-6, IFN-γ, amongst others in a TLR-mediated pathway (Yang et al., 2014). It also mediates generation of mtROS and may recruit autophagic machinery after PAMP recognition in infected macrophages, where *M. tuberculosis* is targeted to autophagosomes in a selective manner (Chauhan et al., 2015). At the same time, classical activation of macrophages in normal or hypoxic condition trigger bacterial killing by inhibiting autophagy, which results in mitochondrial depolarization and ROS generation in AKT dependent manner (Matta and Kumar, 2015, 2016). In the context of *Pseudomonas aeruginosa* activation of NLRC4 inflammasome by mitochondrial damage is checked by mitophagy *in vitro* as well as *in vivo* (Jabir et al., 2015; Harris et al., 2017). Capturing of inflammasome subunits by autophagy is one of the important feature besides mitophagy to suppress inflammation (Harris et al., 2017). In case of gram negative bacterial infection including *Salmonella*, non-canonical inflammasome recognizing LPS promotes inflammation and activation of caspase-11 (Kayagaki et al., 2013). In contrast, selective autophagy is induced when autophagy adaptor Nuclear dot protein 52 kDa (NDP52) recognizes ubiquitin chains on the bacteria, aiding in limiting caspase-11 (Takahama et al., 2018). Similar role of autophagy-mediated control of mitochondrial depolarization, mtROS production and inflammation is on display during Vesicular stomatitis virus (VSV) infection (Tal et al., 2009). Thus, selective autophagy effectively serves as a master regulator in limiting inflammation. Moreover, it is also evident that control of inflammation by autophagy represents the homeostatic arm of selective autophagy, since it is the loss of homeostasis that eventually results in inflammation.

Yet another dimension of cellular homeostasis where autophagy plays a critical role involves extracting energy from catabolic substrates during energy requirement in several stress conditions including bacterial infections. Autophagy-mediated degradation of macromolecules help rebuild new structures and increase basic nutrient pool in the cell. To counteract pathogenic infections, hosts can develop stringent environment to limit the nutrient availability to starve the pathogens (Zhang and Rubin, 2013). For example availability of Fe^2+^ to the pathogens can be restricted in the hosts by molecules like NRAMP and transferrin (Porcheron et al., 2013). Similarly restricting availability of amino acids helps host control bacterial infections (Zhang and Rubin, 2013). While amino acid restrictions imposed by hosts is an important contributor to immunity against pathogens, pathogens could also exploit autophagy to source nutrients. For example in *Leishmania mexicana* autophagy is involved in transferring macromolecules to parasitophorous vacuoles (Schaible et al., 1999). Similarly, Large cell variant form of *Coxiella burnetii* acquire nutrients when autophagosomes fuse with the replicative vacuoles of the bacterium. Similarly, *Chlamydia trachomatis* escapes amino acid limitation in the host by converting itself into an aberrant body form (having less nutritional requirement) from replicating reticulate bodied form (Zhang and Rubin, 2013). *Legionella* with the help of its virulence factor ank B obtain free amino acid which are transferred with the help of host SLC1A5 transporter to the *Legionella* containing vacuole (LCV) containing vacuole. *M. tuberculosis* on the other hand, when challenged by host through tryptophan depletion via IDO expression, is capable of synthesizing tryptophan on its own while residing in the phagocytic vacuole (Zhang and Rubin, 2013). *Francisella tularensis* utilizes non- essential amino acids with ATG-5 independent autophagy (Steele et al., 2013). Interestingly, deprivation of essential nutrients by hosts have helped pathogens to evolve into auxotrophs for up to 10 amino acids thereby remarkably limiting the ability of nutritional immunity of the host (Steele et al., 2015).

Taken together, it is evident that the homeostatic functions of autophagy turns out to be more helpful for bacteria, whether via controlling inflammation or by providing nutrients for the bacteria. This is in contrast from the autophagy-mediated degradation of bacterial pathogens where autophagy works against the pathogen survival. To understand the distinction between the homeostatic arm and anti-bacterial (defense) arm of autophagy, it is important to explore the mechanisms of selective xenophagy regulation as discussed below.

## Bacterial Effectors in Selective Autophagy (Xenophagy) During Infections

Several bacterial effectors are known to regulate selective autophagy through myriad mechanisms. Rapid detection of PAMPs during pathogenic infections is crucial for mounting a strong inflammatory response and control of infection. Therefore, how selective autophagy exhibiting clear anti-inflammatory functions help during bacterial infection? In addition to its homeostatic and anti-inflammatory functions, autophagy can also directly tag bacteria for lysosomal degradation (Gutierrez et al., 2004). This indeed was the earliest understanding that led to the characterization of autophagy as a defense mechanism (Deretic, 2006). Several bacterial effectors are known to impact autophagy regulation in the infected host cells, briefly summarized in Table 1. In case of *Salmonella*, it is known that effectors from Type III secretion system (T3SS) help its escape from *Salmonella*-containing vacuole (SCV) and exposes them to cytosolic PRRs, which results in ubiquitination and recruitment of selective autophagy adaptors like Optineurin (OPTN) and p62 (Herhaus and Dikic, 2017). Recent studies have revealed that *M. tuberculosis* releases its DNA into the cytosol that can be recognized by the Stimulator of IFN genes (STING)-dependent cytosolic sensing pathway, which further aids in marking the bacteria with ubiquitin, and delivering it to the autophagic machinery through the selective autophagic receptors p62 and NDP52 (Watson et al., 2015). This particular process is dependent on the non-conventional secretory system, ESAT-6 secretion system (ESX1) of *M. tuberculosis*, which is also known for its role in mycobacterial virulence. Interestingly, the ESX1 machinery and its effectors like ESAT-6 are known to block the maturation stage of xenophagy selectively (Chandra et al., 2015). In case of *Shigella flexneri*, the virulence factor VirG (also known as IcsA), that is also a ligand for the autophagy protein Atg5, is involved in inducing autophagy (Ogawa et al., 2005), whereas during *Listeria monocytogenes* infection, its toxin listeriolysin (LLO) is responsible for targeting the bacteria to the autophagosomes (Meyer-Morse et al., 2010). Why should intracellular bacteria have virulence mechanisms involving induction of autophagy, a potentially disastrous outcome for the pathogens? It turns out, although intracellular pathogens get targeted via xenophagy for their degradation, they have evolved several mechanisms to inhibit or modulate autophagy at multiple steps in order to survive better in the cell. The manipulation by the bacteria can be done by limiting ubiquitination, by inhibiting the formation of Microtubule-associated protein 1A/1B-light chain 3 (LC3) and Phosphatidylethanolamine (PE) conjugate on the autophagosome membrane or at the stage of autophagosome maturation. For example, *S. flexneri* is able to escape xenophagy by secreting its effector IcsB, which binds competitively to its surface protein VirG, thereby, inhibiting the bacterial recruitment to the phagophore (Ogawa et al., 2005). Additionally, *S. flexneri* effector protein VirA inhibits the activity of Rab1, which is required for early phagosome formation from the ER (Dong et al., 2012). In contrast, *L. monocytogenes* escapes autophagic recognition by ActA protein, which recruits the Arp2/3 complex and Ena/VASP to the bacterial surface for preventing its ubiquitination and autophagic recognition (Yoshikawa et al., 2009). Another virulence factor Inlk of *L. monocytogenes* helps to mask its cell surface by binding to host cytoplasmic Major vault protein (MVP) to block ubiquitination and avoid xenophagy (Dortet et al., 2011). Phospholipases, PlcA and PlcB from *L. monocytogenes* also inhibit autophagy by blocking the lipidation of LC3 (Mitchell et al., 2015). In case of *Salmonella typhimurium*, more than 30 effector proteins are secreted in to the host cytosol via its T3SS2, leading to the recruitment of Focal adhesion kinases (FAK) to the surface of SCV followed by activation of AKT-mTOR and suppression of autophagy (Casanova, 2017). Additionally, the role of effector protein SseL is quite specific in that it deubiquitinates ubiquitin aggregates on the surface of SCV, thereby decreasing its targeting to the autophagosomes (Mesquita et al., 2012). Another effectors from *Salmonella*, SseF and SseG inhibits autophagosome formation by disrupting Rab1-A signaling (Feng et al., 2018). Although, many bacterial proteins are known to disrupt the autophagy pathway indirectly, RavZ, which is a T4SS effector of *Legionella pneumophila* is the only identified bacterial factor that directly inhibits components of the autophagy pathway. RavZ irreversibly cleaves the amide bond linking LC3 to PE, consequently blocking the ability of phagophores to recognize ubiquitylated cargo (Choy et al., 2012). Interestingly, RavZ is not present in all the strains of *L. pneumophila*, therefore they employ *Lp*Sp1 (Sphingosine-1 phosphate lyase), secreted by the Dot/Icm type IV secretion system, which down regulates host shingolipid levels and causes delay in the autophagic response (Rolando et al., 2016). In addition, the autophagy-related SNARE, syntaxin 17, which is recruited to autophagosomes via IRGM, is degraded by the *L. pneumophila* effector Lpg1137 to suppress autophagy and apoptosis (Arasaki et al., 2017; Kumar et al., 2018). Some bacteria are not targeted to the autophagosomes since they are capable of degrading the adaptor proteins. For instance, in the case of Group A *Streptococcus* (GAS), the effector protein SpeB, which is a cysteine protease, degrades the autophagy adaptors p62, NBR1 and NDP52, thereby escaping xenophagy altogether (Barnett et al., 2013). Manipulation of xenophagy by inhibiting the maturation of autophagosomes is well studied in *M. tuberculosis*. The effectors from *M. tuberculosis* ESX-1, a type VII secretion system, inhibit Rab5 to Rab7 conversion on autophagosomes, thus preventing its maturation (Chandra et al., 2015). Another *M. tuberculosis* effector protein, PtpA, a tyrosine phosphatase, inhibits the phagosome-lysosome fusion by phosphorylating vacuolar protein sorting 33B (VPS33B), which is the regulator of membrane fusion (Bach et al., 2008). In addition to this, PtpA also alters the V-ATPase machinery on the phagosome preventing its maturation (Wong et al., 2011). Enhanced intracellular survival (EIS) protein of *M. tuberculosis* also inhibits autophagy by mediating AKT/mTOR pathway via activation of IL-10 (Duan et al., 2016). Some bacteria, instead of inhibiting autophagy, induces it for their own benefit. In this case, augmentation of overall autophagy, rather than promoting bacterial clearance via xenophagy, facilitates the acquisition of nutrients by the invading bacteria. Bacteria like *Anaplasma phagocytophilum, Yersinia pseudotuberculosis, C. burnetii* and *F. tularensis* may sabotage the host defense mechanism elicited by induction of autophagy and the resulting accumulation of autophagosomes via utilizing the autophagic vesicles as nutrient source for microbial growth (Winchell et al., 2016). It is also possible that increased autophagy, through its anti-inflammatory effects, inadvertently helps bacterial survival.

**Table 1:** Bacterial effectors in autophagy and inflammation.

	Bacterial effectors in autophagy		
Bacteria	Ubiquitination/Deubiquitination	Autophagosome targeting/Maturation	Bacterial effectors in inflammation
*Shigella flexneri*		IcsB	lpaH9.8
		Binds to VirG, ligand for ATG5, inhibits phagophore recruitment (Ogawa et al., 2005)	Targets NEMO for degradation (Ashida et al., 2010
		VirA	IpaH 7.8
		Inactivates Rab1 and inhibits the autophagosome formation (Dong et al., 2012)	Activation of NLRC4 inflammasome (Suzuki et al., 2014)
			OspG
			Prevents IkBα degradation, (Kim et al., 2005)
*Salmonella* spp.	SseL	SseF and SseG	AvrA and SseL
	Removes ubiquitin aggregates on	Impairs autophagy initiation via	Prevents degradation of IkBα,
	SCV (Mesquita et al., 2012)	disrupting Rab1 signaling (Feng et al., 2018)	(Rytkonen and Holden, 2007; Ye et al., 2007) SpvD Interferes with the nuclear translocation of p65 and thus NF- αB signaling, (Rolhion et al., 2016)
*Listeria* spp.	Inlk	PlcA and Plc B	InIC
	Mask the bacterial surface by	Inhibits pre autophagosomal maturation	Interacts with IKKα and decreases
	recruiting MVP, prevents ubiquitination (Dortet et al., 2011)	(Mitchell et al., 2015)	phosphorylation of IkB, (Gouin et al., 2010)
	ActA Recruits Arp2/3 complex and Ena/Vasp, prevents ubiquitination (Yoshikawa et al., 2009)		
*Mycobacterium*		Eis	PtpA
*tuberculosis*		Inhibits autophagy by mediating	Partially inhibits NF-B by targeting TAB3
		Akt/MTOR pathway via activation of IL-10 (Duan et al., 2016)	(Wang et al., 2015)
		PtpA Targets VPS33B and V-ATPases to	ESAT-6 Activates NLRP3/ASC inflammasome
		prevent maturation of phagosome	Mishra Bibhuti et al., 2010)
		(Bach et al., 2008; Wong et al., 2011)	
		ESAT-6	
		Prevents Rab5 to Rab7 conversion on	
		autophagosomes, inhibiting maturation	
		(Chandra and Kumar, 2016)	
*Legionella*	SdeA	RavZ	
*pneumophila*	DUB domain contact the ubiquitin	Prevents the lipidation of LC3	
	chains of bacteria	(Choy et al., 2012)	
	(Sheedlo et al., 2015)	*Lp*Sp1,Reduces levels of sphingosine in the cell and further delays autophagic response (Rolando et al., 2016)	

## Bacterial Effectors Impacting Inflammation

During bacterial infection, PAMPs are recognized by the host PRRs like TLRs and Nod-like receptors (NLRs). PAMPs recognition by these receptors activates a proinflammatory response via two major signaling pathways, that are mediated by MAPKs and NF-κB. Inhibition of these pathways is a crucial strategy for bacterial survival in the cell. Many bacterial proteins, such as type III secretion system effectors, toxins, and extracellular adherence proteins, are known to possess anti-inflammatory abilities that helps the bacteria to bypass the host’s response and prolong their survival in the hosts (Table 1). The virulence factors from *L. monocytogenes*, LLO and InlB can activate the NF-κB pathway, whereas its effector protein internalin C (InlC) downregulates the same by directly interacting with IKKα, thereby decreasing the phosphorylation of the inhibitory component of NF-κB, IκB (Gouin et al., 2010). The IKK complex has emerged as the main target of many bacterial effectors in controlling inflammation. The E3 ligase like effector IpaH9.8 of *S. flexneri* also targets IKK. IpaH9.8 mimics host E3 ubiquitin ligases and ubiquitinates NEMO, an upstream component of IKK complex, so as to target it for proteasomal degradation and preventing NF-κB activation (Ashida et al., 2010). Additionally, *S. flexneri* secretes two more E3 ligases, IpaH1.4 and IpaH2.5, which indirectly targets IKK by carrying out the proteasomal degradation of Linear ubiquitin chain assembly complex (LUBAC) (de Jong et al., 2016). LUBAC is a multimeric E3 ubiquitin ligase which is responsible for activating IKK and further NF-κB. The *Salmonella* proteins, SseL and AvrA prevents the degradation of IkBα and thereby impairs NF-kB activation (Ye et al., 2007; Le Negrate et al., 2008). Similarly, OspG, the effector protein of *S. flexneri* prevents IkBα degradation (Kim et al., 2005). *M. tuberculosis* protein PtpA partially inhibits activation of NF-κB pathway by targeting TAB3 (Wang et al., 2015). Sop A, type III secretion system effector of *S. typhimurium* is a HECT type E3 ligase and is reported to target host TRIM 56 and TRIM 65. This leads to the modulation of innate immune receptors RIG-1 and MDA-5, which causes pro-inflammatory cytokine production (Kamanova et al., 2016; Fiskin et al., 2017). Recent studies have shown that *S. typhimurium* effector protein SpvD, a cysteine protease binds the nuclear exportin, Xpo2, resulting in the disruption of normal recycling of importin-α from the nucleus, leading to the defect in nuclear translocation of p65, consequently resulting in the inhibition of NF-κB induced immune responses (Rolhion et al., 2016). During *Yersinia* infection, an acetyltransferase, YopP/J gets translocated into the host cells, thereby acetylating IKK complex as well as MAPK Kinases (MKKs), which prevent their phosphorylation and subsequent inflammatory signaling (Pha and Navarro, 2016). Besides, modulating NF-κB and MAPK signaling, pathogens can also directly restrict or modulate activation of inflammasome. *S. flexneri* utilizes E3 ligase IpaH7.8 to ubiquitylate GLMN protein, which is involved in inhibiting the activation of NLRP3 and NLRC4 inflammasome (Suzuki et al., 2014). *M. tuberculosis* ESAT-6 also potentially activates NLRP3/ASC inflammasome (Mishra Bibhuti et al., 2010). Interestingly, a T6SS effector EvpP from *Edwardsiella tarda* inhibits NLRP3 inflammasome, however, a T3SS effector from the same pathogen activates NLRC4 and NLRP3 inflammasomes (Chen et al., 2017). It is important to note that inflammation and resulting cell death in itself can impact bacterial pathogens survival within the host. Considering autophagy as an anti-inflammatory process, it is possible that bacteria may strive to inhibit inflammation through activation of autophagy. Immune cells like macrophages acquire inflammatory and microbicidal properties by inhibiting autophagy under the influence of pro-inflammatory environment (Matta and Kumar, 2015, 2016). However, knowing that autophagy can also directly target bacterial pathogens for lysosomal degradation, how a possible selectivity may playout during infections that allows bacteria at one hand to escape autophagic targeting while homeostatic arm of autophagy continues unabated is an interesting question. A clue to such selectivity arises from our understanding of the autophagy adaptors, which are critical for selective autophagic targeting of various cargos. Curiously, there are only a handful of autophagy adaptors known so far and their recruitment/role in homeostatic autophagy or xenophagy are very much overlapping, leaving the question open that how selectivity is ensured.

## Autophagy Adaptors: Shared Player for Selective Autophagy During Infection and Inflammation

**FIGURE 2:**
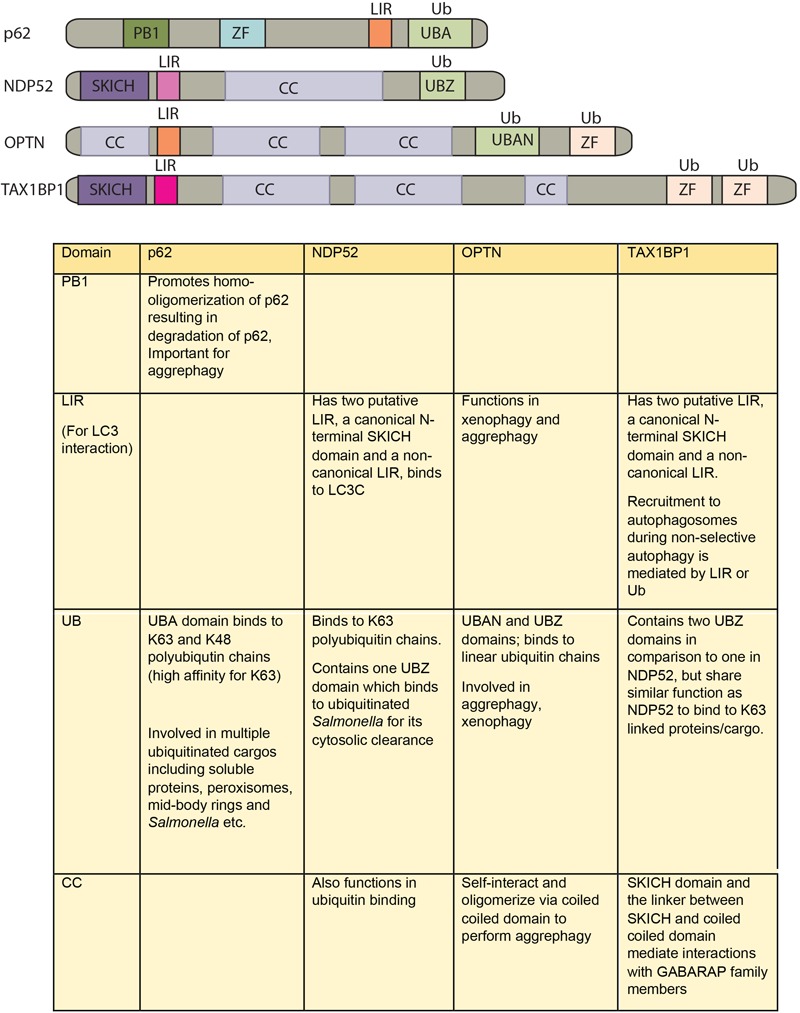
Domain structure of autophagy adaptors and their function. The following abbreviations are used for each domain: PB1, Phox and Bem1 domain; CC, coiled-coil domain; LIR, LC3-interacting region; UBA, ubiquitin-associated domain; SKICH, SKIP carboxyl homology domain; ZF/UBZ, Ubiquitin binding Zinc-finger domain; UBAN, ubiquitin binding in ABIN and NEMO domain.

**FIGURE 3:**
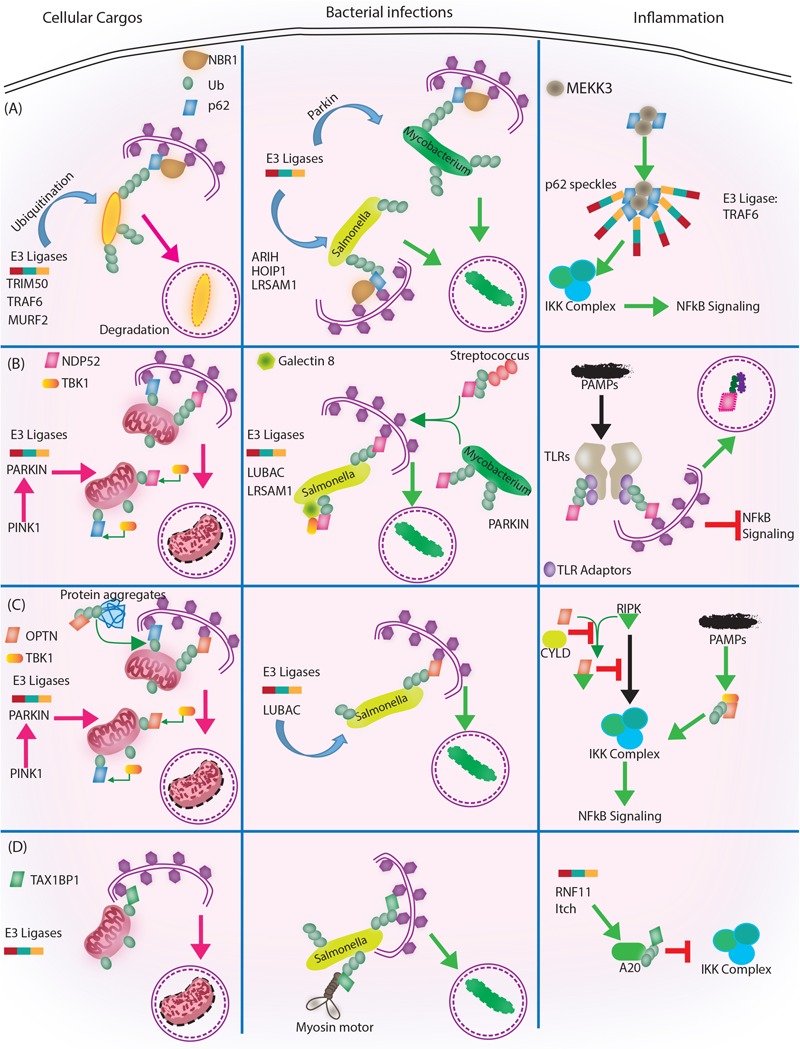
Autophagy adaptors in selective autophagy, xenophagy and inflammation. Roles of different autophagy adaptors are highlighted here in the context of selective autophagy of intracellular cargos, xenophagy and inflammation. **(A)** p62/SQSTM1: E3 ligases TRIM 50, TRAF 6, MURF 2 ubiquitinate the intracellular cargos, followed by their recognition by autophagy adapter p62 and NBR1. Adaptors then target the cargo to autophagosome for subsequent degradation. During bacterial infection, p62 adaptor protein recognizes targets that are tagged by E3 ligases like ARIH, HOIP1, LRSAM1 (which act on *Salmonella*), after which p62 and NBR1 are recruited to the bacteria and targets *Salmonella* for autophagic degradation. In case of *M. tuberculosis* so far only Parkin has been shown to act as E3 ligase leading to K63 ubiquitination, p62-NBR1 recruitment and targeting of *M. tuberculosis* to autophagosomes. Finally, p62 is directly implicated in regulating inflammation. The PB1 domains of p62 homo and heterodimerize while interacting with MEKKK3. This complex further co-localizes to TRAF 6 oligomers, forming what is known as p62 speckles. This complex then phosphorylates and ubiquitinates IKK complex, inhibiting NFκB signaling. **(B)** NDP52/CALCOCO2: Upon mitochondrial damage the recruitment of PINK1/PARKIN E3 ligase help in ubiquitinating mitochondria and activating TBK1, which subsequently phosphorylate both NDP52 and p62 Ubiquitination of mitochondria and tagging with p62 and NDP52 helps in targeting mitochondria to autophagosome. During *Salmonella* infection, LUBAC complex and LSRAM1 E3 ligases ubiquitinate the bacteria. Phosphorylation of NDP52 by TBK1, help tagging of the bacteria with NDP52. Here cytosolic Galectin 8 also takes part in the process and interacts with NDP52. For recruitment of *M. tuberculosis* parkin mediates ubiquitination of *M. tuberculosis*. NDP52 is also recruited to *M. tuberculosis* and targets it to autophagosome. Rab35 and NDP52 also mediate targeting of *Streptoccocus* to autophagosomes. Bacterial PAMPS are recognized by TLR followed by recruitment of TLR adapters. The TLR adaptors get ubiquitinated and recognized by autophagy adapter NDP52. Along with the TLR adaptors, autophagy adaptors undergo degradation via autophagosome maturation called adaptophagy and controls inflammation. **(C)** OPTN: OPTN acts in mitophagy in a manner very similar to NDP52 where PINK1 and PARKIN E3 ligases are activated and recruited to mitochondria for ubiquitination. Ubiquitinated mitochondria are recognized by OPTN for targeting them to autophagosomes. In addition to mitophagy for degrading intracellular cargo OPTN performs aggrephagy as well. During bacterial infections, OPTN’s role has been shown in the context of LUBAC complex mediated K63 and M1 polyubiquitination of *Salmonella*. Upon recruitment, OPTN targets the bacterium to autophagosomes for degradation. OPTN is also shown to inhibit IKK complex by interacting with RIPK. Interaction of OPTN with a deubiquitinating enzyme called CYLD, which deubiquitinates OPTN and RIPK allows RIPK mediated activation of IKK complex and inflammation. Similarly, PAMPs can activate TBK1, which gets autophosphorylated and subsequently binds to TBK binding domain of OPTN to alleviate inflammation. **(D)** TAX1BP1/CALCOCO3: Similar, to other autophagy adapters like NDP52 and OPTN TAX1BP1 also performs mitophagy where E3 ligases are not very well known, however, Parkin is the most likely candidate. In *Salmonella*, TAX1BP1 interacts with myosin motor VI and induces autophagosome and lysosome fusion subsequently helping in xenophagy of polyubiquitinated *Salmonella*. TAX1BP1 interacts with A20, an NFκB inhibitor to control inflammation via inhibiting IKK complex. Here, RNF 11 and Itch E3 ligases helps recruitment of TAX1BP1 to A20 for autophagic targeting. While we have used very selected examples to highlight the functional overlaps between different autophagy adaptors during selective autophagy, it must be noted that inhibition of selective autophagy like mitophagy also contributes to inflammation. This figure therefore showcases the complex regulatory events and points toward the existing lacunae in our understanding of selective autophagy, especially in the context of bacterial infection and inflammation.

How different cargos (intracellular or pathogenic) are selectively targeted for autophagic degradation? One of the first steps involves tagging of the cargo by ubiquitin chains through the action of E3 ubiquitin ligases. Each of the cargos destined for autophagic degradation must get ubiquitinated for recognition by autophagy adaptor molecules. Autophagy adaptor proteins serve as a bridge between the cargo to be degraded and the nascent autophagosomes. All adaptor proteins share three common domains – LC3 interacting region (LIR) domain (through which they interact with LC3II decorating the autophagosomes), dimerization or multimerization domain and ubiquitin-binding domain (Behrends and Fulda, 2012). In addition to their role in selective autophagy, these proteins also regulate innate immunity signaling pathways, thus representing a new class of PRRs, the sequestosome-1-like receptors (SLRs) causing inflammation (Deretic et al., 2013). In the sections below, we discuss four key autophagy adaptors p62, NDP52, OPTN and TAX1BP1 in detail including their domains, interacting partners as well as their involvement in regulating autophagy under diverse contexts, which is also summarized in Figures 2, 3.

## P62

p62 is among the first mammalian autophagy adaptors initially identified and described. The Phox and Bhem 1 (PB1), LIR, and Ubiquitin associated (UBA) domains of p62 are implied in the degradation of ubiquitinated cargos by selective autophagy. By directly interacting with several E3 ligases, such as TRIM50, TRAF6 and MURF2., which promotes ubiquitination of p62 substrates, it is involved in the formation of inclusion bodies and execute aggrephagy (Rogov et al., 2014). Interestingly, p62 also contributes to pexophagy (Kim et al., 2008; Tripathi et al., 2016) and mitophagy, which is dependent on E3 ligase-“Parkin” (Geisler et al., 2010). The importance of p62 in executing anti-bacterial autophagy or xenophagy is primarily explored in controlling the invading bacteria *S. typhimurium* (Zheng et al., 2009). Other bacterial species such as *S. flexneri* and *M. tuberculosis* are also reported to be selectively targeted in a p62-dependent manner for recruitment and delivery into nascent LC3-positive isolation membranes for autophagic degradation (Mostowy et al., 2011; Franco et al., 2017). In *ex vivo* infection experiments using macrophages, it is shown that p62 co-localizes with *M. tuberculosis* and controls its survival and replication (Sakowski, 2015). True to the above observation, knocking down p62 in macrophages during *ex vivo* infections increases *M. tuberculosis* survival. However, the redundancy in the p62-mediated physiological functions gets evident *in vivo* in mice, where *p62^-/-^* mice never show any sickness past 80 days of *M. tuberculosis* infection and effectively controls bacterial replication (Kimmey et al., 2015). Additional modulators of p62, which functions in regulating xenophagy have been reported. For example, TBK1 plays an important role in promoting the xenophagy by activating p62 via phosphorylation of Serine 403 in the UBA domain of p62. UBA domain phosphorylation strongly enhances the activity of p62 and is implicated in the elimination of *M. tuberculosis* via autophagy (Pilli et al., 2012). Additional mechanisms for p62-mediated xenophagy are also reported, like involvement of the lysosomal protein DRAM-1 in recruiting p62 for restricting *M. marinum* infection in zebrafish (Meijer and van der Vaart, 2014).

Considering their critical role in regulating selective autophagy, adaptors like p62 could have direct involvement in controlling inflammation in a selective manner. The structural speckles of p62 are said to be involved in TRAF6 oligomerization resulting in NF-κB activation, and subsequent inflammation (Nakamura et al., 2010). On the contrary, the anti-inflammatory functions of p62 are also well known, for example, it down-regulates inflammation in response to adiponectin after LPS stimulation (Tilija Pun and Park, 2017). p62 is also shown to regulate oxidative stress. Activated TAK1 phosphorylates p62, which induces the interaction of p62 with keap-1 that causes subsequent degradation of keap-1 through autophagy (Hashimoto et al., 2016). This results into increased Nrf2 expression, the master regulator of antioxidant gene expression. Since, TAK1 participates in TLR, NLR, IL-1 and stress induced pro-inflammatory signaling, its regulation of p62-Keap-1-Nrf2 axis characterizes a link between inflammation and redox homeostasis (Hashimoto et al., 2016). In addition to TAK-1, activated mTOR pathway and TLR-induced TBK-1 are also involved in inducing the interaction of p62 with Keap-1 (Ichimura et al., 2013). Although, p62 is involved in inflammation as well as in autophagy, its role in inflammation during intracellular infection is still elusive and needs further explorations.

## NDP52

NDP52 is an important selective autophagy adaptor primarily performing mitophagy to maintain cellular homeostasis by removing damaged mitochondria from the cell (Heo et al., 2015). It is also found to play a significant role in xenophagy, as confirmed by several studies. It targets various bacteria including *Streptococcus pyogenes, Salmonella enterica* and *S. flexneri* to autophagy for their selective degradation (von Muhlinen et al., 2010; Mostowy et al., 2011; Minowa-Nozawa et al., 2017). Since, there is a substantial amount of functional redundancy found in autophagy adaptors, it results into the recruitment of two or more adaptors to the same bacterium. Additionally, few reports also highlight the fact that the same adaptor can induce targeting of different bacteria to the intrinsically different autophagosomes. For instance, p62 and NDP52 targets *Shigella* to autophagosomes in actin-septin dependent manner, whereas p62 and NDP52 are recruited independently to *Listeria* to target them into autophagosome via actin-septin independent pathways (Mostowy et al., 2011). This reinforces the view that different bacterial pathogens can evoke different pathways of selective autophagy and therefore it needs to be further investigated. Although xenophagy is aided by several cargo receptors, it is important to note that the recruitment of adaptors is independent of each other and they bind to different micro domains of bacteria (Cemma et al., 2011). NDP52 is able to interact with all the human ATG8 orthologs, but it selectively binds to LC3C via its non-canonical LIR (CLIR) domain to perform the antibacterial activities (von Muhlinen et al., 2012). Parkin, a well-known E3 ligase ubiquitinates NDP52 for mitophagy in a TBK1 phosphorylation dependent manner (Heo et al., 2015). Parkin is also involved in the recruitment of NDP52 to *M. tuberculosis* phagosomes upon infection, as the knockdown of *Park2* decreases the co-localization of *M. tuberculosis* with NDP52, p62 as well as NBR1 (Manzanillo et al., 2013). Besides ubiquitin, cytosolic Galectins also play an important role in cargo targeting to the autophagosome. NDP52 is shown to bind to Galectin 8 for removal of *Salmonella* via selective autophagy (Thurston et al., 2012). Besides, ubiquitination and phosphorylation there are few other proteins, which might show interaction with autophagy adaptors to activate them. Recently, Rab35 has been reported to control Group A *Streptococcus* (GAS) degradation by xenophagy via recruiting NDP52 (Minowa-Nozawa et al., 2017). However, Rab35 involvement in autophagy is not only restricted to xenophagy, but also in mitophagy, starvation-induced autophagy, both of which occur via recruiting NDP52 (Minowa-Nozawa et al., 2017).

NDP52 also take part in reducing inflammation via down-regulating the NF-κB signaling. In a sequencing study of CD patients versus healthy controls, whole exome sequencing of 42 CD patients revealed an interesting mechanism. A missense mutation Val248Ala in NDP52 failed to recognize polyubiquitinated adaptors resulting in high NF-κB activity, thereby causing more inflammation in CD patients. Importantly, the study also highlights the importance of NDP52/CALCOCO2 adaptophagy (Ellinghaus et al., 2013; Till et al., 2013), where the turnover of autophagy adaptors plays an important role in regulating inflammation.

## OPTN

Optineurin (OPTN) is a 67 KDa intracellular protein found in different tissues and has various domains like C-terminal zinc finger, leucine zipper domain, a LIR domain, and ubiquitin binding in ABIN and NEMO domain (UBAN) domain (Kim et al., 2016; Slowicka and van Loo, 2018). It also consists of coiled-coil motifs, which mediates its oligomerization. It is an important autophagy adaptor as demonstrated by its role in mitophagy, aggrephagy as well as xenophagy (Wild et al., 2011; Heo et al., 2015; Richter et al., 2016; Slowicka and van Loo, 2018). The role of OPTN in mitophagy was established in studies, which show that mutations in OPTN are associated with amyotrophic lateral sclerosis (ALS) and glaucoma due to mitochondrial dysfunction (Wong and Holzbaur, 2014). Studies have also shown that OPTN is required to restrict the growth of *S. enterica* upon infection (Wild et al., 2011). The key player in OPTN-mediated selective autophagy is TBK1, which phosphorylates it within LIR domain at Ser-177 and enhances its activity (Richter et al., 2016). Although, mitophagy and xenophagy are mediated by OPTN in ubiquitin-dependent manner, aggrephagy can be both dependent as well as independent of ubiquitin. It has been reported that OPTN recognizes targets like mutant SOD-1 and huntington protein (associated with ALS and Huntington’s disease respectively) by an ubiquitin-independent pathway (Korac et al., 2013).

OPTN majorly inhibits inflammation by negatively regulating NF-κB signaling (Nagabhushana et al., 2011; Slowicka and van Loo, 2018). It is capable of competing with NEMO, to bind to RIPK1, a major factor for activating NF-κB, thereby resulting in down-regulation of NF-κB (Zhu et al., 2007). OPTN is also found to interact with a deubiquitinase enzyme CYLD, leading to deubiquitination of NEMO and RIPK1, thereby aiding in inhibition of TNF mediated NF-κB activation (Nagabhushana et al., 2011). However, role of OPTN *in vitro* is in contrast to the *in vivo* findings where OPTN knock out or knock in mice does not affect NF-κB signaling (Munitic et al., 2013; Slowicka et al., 2016). OPTN associated mitophagy have been extensively described, more recently it is shown to remove protein clusters associated with ER called aggrephagy (Tschurtschenthaler and Adolph, 2018). It might be further implicated to regulate inflammation since unused proteins are deleterious to the host cell. A decrease in the expression of OPTN has been correlated with the patients of CD. The reduced expression of OPTN was linked to a genetic variation due to SNP (rs12415716) in a subset of CD patients’ cohort that was examined (Smith et al., 2015). In addition, OPTN also limits persistent ER-Stress. In intestinal cell, it targets IRE1-α degradation to combat the ER based inflammatory response (Tschurtschenthaler et al., 2017). On the contrary, OPTN mediated activation of IRF3 results into type 1 IFN production ultimately leading to bacterial clearance (Slowicka et al., 2016). As is evident here, the functional versatility of OPTN makes it an important autophagy adaptor.

## TAX1BP1

TAX1BP1/CALCOCO3 is a close paralog of NDP52/ CALCOCO2. Its role in xenophagy was first highlighted in the study that shows its involvement in the removal of *S. typhimurium* (Tumbarello et al., 2015). The clearance of the bacteria is dependent on the binding of TAX1BP1 to the myosin motor VI, which aids in the fusion of autophagosomes with the lysosomes (Tumbarello et al., 2015). Besides bacteria, the role of autophagy adaptors is also reported during viral infection. For instance, both TAX1BP1 and NDP52 can impact the replication of Measles Virus (MeV) in the cells by interacting with the MeV proteins and facilitating the maturation of autophagosomes (Petkova et al., 2017). These functions, are however, independent of their potential role in NFκB signaling. Surprisingly, knocking down OPTN using siRNAs does not have any effect on the MeV replication suggesting that there is specificity/selectivity among the adaptors to interact with different viral proteins (Petkova et al., 2017).

In an uninfected Streptozotocin (STZ)- induced diabetic mice model, TAX1BP1 overexpression in the heart attenuates inflammation, oxidative stress, and apoptosis that results in improved cardiac function (Xiao et al., 2018). It has been shown to interact with ubiquitin-editing enzyme A20, regulating NFκB and IRF3 signaling thereby controlling inflammation to increase the longevity of host cells (Shembade et al., 2007). The E3 ligase Itch and RNF11 recruitment to A20 is shown to be dependent on TAX1BP1 that restricts pro-inflammatory state in the cell (Shembade et al., 2009). Similarly, in RNA virus infection it controls RIG-1/MDA-5 mediated production of IFNβ (Parvatiyar et al., 2010; Choi et al., 2017). Few reports also manifest anti-inflammatory roles of TAX1BP1 during other viral infections. For example, dysregulation of TAX1BP1 in HTLV-1 infection can result in the induction of diverse forms of inflammatory disorders (Nakano et al., 2013). Recently, TAX1BP1 has been shown to play an important role in negatively regulating the VSV and Sendai virus associated apoptosis, as it gets degraded upon viral infection (Choi et al., 2017). Degradation of TAX1BP1 increases apoptosis and this could help in limiting the prolonged antiviral state of the cells. TAX1BP1 can also translocate to the mitochondria and interact with MAVS. This helps the recruitment of E3 ligase Itch to MAVS for its ubiquitination and degradation and thus restricting virus mediated apoptosis (Choi et al., 2017). Although, the anti-inflammatory roles of TAX1BP1 in uninfected and virus infected cells are well known, studies demonstrating its role in controlling bacterial associated inflammation remain unexplored.

## Ubiquitin Ligases and Deubiquitinases in Intracellular Bacterial Infections

One common theme across all the autophagy adaptors discussed above is ubiquitination of the cargos for subsequent adaptor binding. Whether the target is intracellular pathogens like *Salmonella, Lysteria* and *Mycobacteria* or cellular cargos like damaged mitochondria, ERs, peroxisomes or selective proteins; all of them must get ubiquitinated before they are recognized by the autophagy machinery. Ubiquitination of the cargos requires subsequent action of three enzyme cascades- ubiquitin activating (E1), ubiquitin conjugating (E2) and ubiquitin ligase (E3). In humans, nearly 2 E1s, about 40 E2s and more than 600 E3 ligases are known. Since, E3 ligases are the most heterogeneous, they can mediate substrate specificity (Morreale and Walden, 2016) As far as proteins are concerned, ubiquitin chain length and ubiquitin linkage may impact the capacity of ubiquitinated proteins to get targeted for autophagy. Proteins which are coated with K-63 linked ubiquitin chains are mainly cleared through autophagy (Linares et al., 2013), whereas those which are decorated with K-48 or K-27 ubiquitin chains are targeted for proteasomal degradation (Grumati and Dikic, 2018). The UBA domain of p62 shows more affinity toward K-63 chains, even though it binds both K-63 as well as K-48 chains (Long et al., 2008). Similarly, M1-linked polyubiquitin chains on the bacteria attracts OPTN more (Noad et al., 2017). Several E3 Ligases are known which ubiquitinates cytosolic targets as well as bacteria and target them for selective autophagy. Parkin is one of the most studied E3 ligase and is mainly responsible for the ubiquitination of a plethora of mitochondrial membrane proteins and thus is involved in mitophagy (Heo et al., 2015). In addition to mitophagy, Parkin is also involved in ubiquitination of *M. tuberculosis* and targeting it to the autophagosomes via p62 and NDP52 receptor proteins (Manzanillo et al., 2013). Interestingly, Smurf-1, a newly identified ubiquitin ligase polyubiquitinates not only *M. tuberculosis* but also *L. monocytogenes* (Franco et al., 2017). Moreover, smurf-1 seems to work synergistically with Parkin, since BMDMs from Smurf-1^-/-^, Parkin^-/-^ double knockout animals supported enhanced replication of *M. tuberculosis* in comparison to single knockouts (Franco et al., 2017). Similar co-operativity was also observed during *in vivo M. tuberculosis* infection in mice. Additional E3 Ligases responsible for the pathogen ubiquitination are LRSAM1, ARIH, HOIPI and LUBAC complex. However, the ubiquitination pattern deployed by these enzymes may differ. LRSAM is known for forming K6 and K27 chains on the surface of *Salmonella*, whereas, ARIH forms K48 chains and HOIP1 is involved in linear ubiquitination (Huett et al., 2012; Noad et al., 2017; Polajnar et al., 2017). It has been reported that M1-linked polyubiquitination on the bacterial surface recruits OPTN via E3 ubiquitin ligase complex LUBAC. However, the recruitment of p62 and NDP52 occurs independently of LUBAC, demonstrating that the functions of few adaptors are not completely redundant (Noad et al., 2017; Slowicka and van Loo, 2018). During *Salmonella* infections, TRIM32, an E3 ligase, interacts with TRIF and adds a layer of complexity in its selective degradation in TAX1BP1 dependent manner. Here, deficiency of TAX1BP1 leads to inhibition of degradation of TRIF thereby turning off TLR3/4 mediated innate immune responses and inflammation (Yang et al., 2017). Other E3 ligases like RNF166 is found to be a key protein that controls the recruitment of ubiquitin as well as autophagy adaptors to *Salmonella*, by catalyzing K29- and K33-linked polyubiquitination of p62 (Heath et al., 2016). However, ubiquitination of bacteria by RNF166 is not studied. On the similar line, another protein UBQLN-1, consisting of ubiquitin like domain, an UBA domain and a STl1 motif, targets *M. tuberculosis* to autophagy after recruiting ubiquitin, p62 and LC3 (Sakowski et al., 2015). Many of the E3 ligases like LRSAM1 which are known to ubiquitinate other bacterial pathogens are unable to ubiquitinate *M. tuberculosis*. It is possible that while they may still get ubiquitinated, they could recruit certain deubiquitinase (DUBs) to strip themselves of ubiquitin tags and mask from the autophagy adaptors.

DUB’s are the proteins, possessed mainly by the host cells, to execute deubiquitination. Besides, deubiquitinating intracellular cargos, these DUB’s may also target intracellular pathogens for deubiquitination. DUB’s are mainly present in all eukaryotic cells and almost more than 100 of them have been discovered in humans covering important regulatory functions of the cells. Since ubiquitination helps in the degradation process of pathogens including bacteria and viruses, many pathogens have evolved DUB’s or DUB’s like molecules so as to interfere with the host ubiquitination process. For example, in *C. trachomatis*, Chla1 and Chla2 have been reported to hydrolyse ubiquitinated and neddylated substrates. *C. pneumonia* has an Otubain like effector (OTU) called as ChlaOTU, having deubiquitinating activity which can cleave K63 and K48 linked polyubiquitin chains of the target/cargo (Sheedlo et al., 2015; Pruneda et al., 2016). It is also reported that NDP52 and ChlaOTU interacts at the bacterial entry site to reduce ubiquitin accumulation. Similarly, in *Shigella* and *Rickettsia*, ShiCE and RickCE function as deubiquitinating enzymes and prefer K63 linked targets (Pruneda et al., 2016). Structural analysis of DUB domain of Sde A in *L. pneumophila* revealed its molecular contacts with ubiquitin on bacteria containing phagophore. Importantly, unlike other eukaryotic counterparts, SdeA_Dub_ recognizes Glutamine 40 patch of ubiquitin rather than Isoleucine 44 on bacterial phagosome (Sheedlo et al., 2015; Pruneda et al., 2016). Sid E effector family of *L. pneumophila* which remain involved in ubiquitination of the target substrates, therefore help bacteria to replicate in amoeba. This Sde E effector family also contain a DUB domain which help in reducing the ubiquitin level on LCV (Sheedlo et al., 2015). Besides bacteria, certain viruses like Herpes Simplex virus-1 (HSV-1) has UL-36, pertaining deubiquitinase activity responsible for deubiquitinating TRAF-3 (Wang et al., 2013). This activity is reported to remain conserved in Epstein Barr Virus and Cytomegalovirus. Interestingly, there are cases where in few pathogens the same enzyme performs two or more functions attributing to redundancy in enzyme functionality. *Yersenia* virulence factor Yop J, is an acetyl transferase and also contributes in deubiquitinating Iκβ and limit NFκβ induced inflammation (Rytkonen and Holden, 2007; Danelishvili et al., 2014). Intriguingly, most of the bacterial DUB’s discovered so far have been shown to interfere with the host ubiqutination or deubiquitination pathways. Studies regarding their interaction with specific autophagy adapters which might lead to rescue of these pathogens from selective degradation remain unexplored. Interestingly, some pathogens may also modulate functions of host DUBs to help them evade xenophagic targeting. For example in a genome-wide siRNA knockdown study USP9Y, a deubiquitinase, was shown to help intracellular *M. tuberculosis* survival (Kumar et al., 2010). In another recent study, OTULIN, a host DUB, specifically deubiquitinates M1 linked ubiquitin chains, thus maintaining balance of conjugation and deconjugation of ubiquitin chains on *Salmonella* (van Wijk et al., 2017). Knocking down OTULIN in the cells results in increased inflammation and *Salmonella* restriction (van Wijk et al., 2017)

## Future Perspectives

We emphasized in this review that autophagy is a cellular homeostatic function, which gets co-opted during various stress conditions including bacterial infections. Generation of pro-inflammatory state upon bacterial infections is a common antibacterial strategy adopted by the host. At the same time, activation of autophagy can also help to directly target the intracellular pathogens for degradation via xenophagy. Within an infected cell, where both inflammatory responses and anti-bacterial autophagy could occur simultaneously, what ensures the selectivity and balance between the two different arms of autophagy? This question is pertinent during pathogenic conditions since as discussed above, the cellular machinery involved in regulating autophagy for homeostasis, inflammation or xenophagy is more or less constant. Thus adaptors like p62, OPTN, TAX1BP1 and NDP52 could be recruited to different cargos including bacterial targets in varying proportions for subsequent targeting to autophagosomes. With the examples discussed in the above sections and many more which could not be discussed due to space constraints, it is plausible that while the adaptors are necessary for selective autophagy, they themselves are not solely responsible in ensuring selectivity of cellular cargos for degradation. This part is also depicted in the schematic shown in the figure summarizing these events (Figure 3). In the view of the above, the role of ubiquitin ligases becomes most critical in recognizing the autophagy cargo and subsequently tagging them for degradation. Questions like involvement of kind of ubiquitination like degree (mono or poly-ubiquitinatred) and linkage (K48, K63, K11, K27 etc.) in deciding the fate of cargo could also emerge in the future. The foundation for such selectivity is already available, given the distinction in fate of K48 versus K63 ubiquitin chains among many others. Similarly, several studies also point to distinctiveness originating due to the degree of ubiquitination like mono-, oligo- or poly-ubiquitination (Kwon and Ciechanover, 2017). To add further complexity, there are also reports suggesting role of autophagy independent of adaptors or ubiquitination. For example, *M. smegmatis*, a non-virulent strain of mycobacterium is degraded by LC3 targeting without involving membrane damage and ubiquitination. Here bacterial killing inside macrophages involves activation of autophagy via a TLR2-dependent mechanism in a p62 and NDP52 independent manner (Bah et al., 2016). Similarly, *M. marinum* ESX-1 secretion system is implicated in LC3 dependent phagocytosis but not ubiquitination (Bah et al., 2016). A better understanding of selective E3 ligase recruitment, activation and downstream recruitment of adaptors followed by activation across cargos like intracellular pathogens as well as cytosolic cargos during infection constitutes a major area of investigation in future. Such studies may yield clearer picture of how the observed selectivity and specificity in maintaining the equilibrium between homeostatic and defense arm of autophagy is ensured in the cells.

## Author Contributions

VS, SV, SS, and DK wrote the manuscript. VS, SV, ES, SS, and DK reviewed the manuscript.

## Conflict of Interest Statement

The authors declare that the research was conducted in the absence of any commercial or financial relationships that could be construed as a potential conflict of interest.
